# The Effects of Whole-Body Vibration Exercise on Anticipatory Delay of Core Muscles in Patients with Nonspecific Low Back Pain

**DOI:** 10.1155/2021/9274964

**Published:** 2021-08-03

**Authors:** Yi-Li Zheng, Hao-Yu Hu, Xiao-Chen Liu, Xuan Su, Pei-Jie Chen, Xue-Qiang Wang

**Affiliations:** ^1^Department of Sport Rehabilitation, Shanghai University of Sport, Shanghai, China; ^2^Department of Rehabilitation Medicine, Shanghai Shangti Orthopaedic Hospital, Shanghai, China; ^3^Department of Rehabilitation Medicine, Shanghai East Hospital, Shanghai, China

## Abstract

**Objective:**

The objective of this study is to determine the effect of whole-body vibration (WBV) exercise on the anticipatory delay of core muscles in nonspecific low back pain (NSLBP) patients.

**Methods:**

Forty participants with NSLBP were randomly divided into the WBV group and the control group. The sEMG signals of deltoid, erector spines (ES), multifidus (MF), rectus abdominis (RA), and transversus abdominus/internal oblique muscles (TrA/IO) were recorded before and after the intervention in the weight-shifting task. The relative activation time of each muscle was calculated.

**Results:**

In the WBV group, the relative activation time of bilateral MF and bilateral TrA/IO was significantly reduced on shoulder flexion (right MF: *P*=0.014; left MF: *P*=0.011; right TrA/IO: *P*=0.008; left TrA/IO: *P*=0.026). As for shoulder abduction, except for the left TrA/IO and the left RA, the relative activation time of other muscles was significantly reduced (right ES: *P*=0.001; left ES: *P* < 0.001; right MF: *P*=0.001; left MF: *P*=0.009; right TrA/IO: *P* < 0.001; right RA: *P*=0.001). In the control group, there was no significant difference in the relative activation time of each muscle before and after the intervention (*P* > 0.05).

**Conclusions:**

WBV exercise can effectively alleviate the anticipatory delay of core muscles in NSLBP patients, but the long-term effects still need further study. This trial is registered with ChiCTR-TRC-13003708.

## 1. Introduction

Nonspecific low back pain (NSLBP) is the most frequent form of low back pain. The proportion of NSLBP in low back pain accounts for up to 90% [1]. Because NSLBP has an unknown pathoanatomical cause, treatment concentrates on reducing pain and its consequences [2]. There is plenty of evidence demonstrating that NSLBP prominently impacts on postural control [3–5], hypothesizing that altered postural control may overload the passive tissues of the spine, contributing to low back pain symptoms [2, 6].

Anticipatory postural adjustments (APAs), happening ahead of voluntary functional movements, are the essential aspects of postural control [7] and seen as a key role to maintaining lumbopelvic stability [8]. Before the onset of predictable postural movement, patients with low back pain reported delayed feedforward activation of deep abdominal muscles [7]. In addition, the alteration of lumbar paraspinal muscle activity occurring in patients with low back pain gives rise to changes in not only the nervous system including reflex inhibition and muscle's nerve supply loss but also supraspinal changes [9, 10]. Surface electromyography (sEMG) is a clinical tool recording electric activities of lumbar muscles in both static and dynamic postures [11]. The relative activation time of muscles in response to expected perturbations, as a measure of APAs, has been developed to be an attempt to explore and expand the clinical utility of sEMG in the field of NSLBP. Studies have shown that the altered activity of the lumbar spinal muscles, for example, erector spinae (ES) and lumbar multifidus (MF), is thought to cause NSLBP or may be secondary to an episode of low back pain [12, 13]. Researchers have shown an anticipatory delay of MF in those with a history of NSLBP [14] and in those with experimentally induced low back pain [15]. This indicates that the recruitment of lumbar spinal muscles is altered in low back pain patients, furthermore, potentially reducing its effectiveness during rapid arm lifting. Also, it has been established in the experiments that low back pain has shown anticipatory delay in stabilizing muscles such as the ES [16] and transverse abdominis (TrA) [17]. Sadeghi et al. [18] investigated the timing of the activation of lumbar muscles, including transverse abdominus/internal oblique (TrA/IO), ES, and rectus abdominis (RA) and demonstrated that TrA/IO has a significant onset delay during unilateral rapid arm movements. Hodges et al. [16] assessed the EMG activity in the superficial and deep fibers of the MF during functional tasks and observed delayed muscular activation during induced pain.

Among our previous studies, a cross-sectional study demonstrated a negative correlation between lumbar neuromuscular function and pain in NSLBP patients [19] and a meta-analysis verified that compared with general exercise, core stability exercise is more effective in alleviating pain and increasing the lumbar muscular functional status in patients with low back pain in the short term [20]. As a new type of core stability exercise in pain relief [21], whole-body vibration (WBV) exercise requires the individual to perform static or steadily controlled exercises on an oscillating platform [22, 23] and becomes a credible procedure for enhancing muscular performance [24–27]. A number of vibration-related research studies have suggested that these positive acute effects are attributed to neural adaptation, containing increased facilitated stretch reflex and muscle activation [23, 28, 29]. It is worth noting that the parameters used in WBV could influence the nervous system's neuromuscular responses. High vibration levels for prolonged periods of time increase the risk of low back pain [30, 31], but it has been documented that frequencies below 20 Hz plus exercise intervention enhanced NSLBP patients' lumbar segmental stabilization [32] and proprioception [33]. However, less is known about whether the delayed activation of lumbar muscles is altered after WBV exercise during the weight-shifting task which is induced by upper extremity lifting.

A better understanding of how WBV exercise affects the NSLBP patients' lumbar muscles APAs during functional tasks may help study the neuromuscular disfunction commonly encountered clinically. As such, we conducted this study as an extension of our previous study [33] to further evaluate the acute effects of WBV exercise on anticipatory delay of core muscles in NSLBP patients.

## 2. Materials and Methods

The study was a single-blind randomized controlled study and approved by the Ethics Committee of the Shanghai University of Sport, China, and by the Chinese Clinical Trial Registry (registry number ChiCTR-TRC-13003708). All participants signed written informed consent. As such, we conducted this study as an extension of our previous study [33] to further evaluate.

### 2.1. Sample Size

Use GPower 3.1.9.2 to count power calculation. Previous studies reported that the effect size of the transversus abdominus/internal oblique (TrA/IO) was 0.957 after 4 weeks of ordinary physical therapy for low back pain [34]. Therefore, to conduct a paired-samples *t*-test, with an alpha value of 0.05 (2-tailed), power of 0.95, and effect size of 0.957, the estimated sample size was 17 participants; that is, the sample size required for the study was 17 participants.

### 2.2. Participants

**Figure 1:**
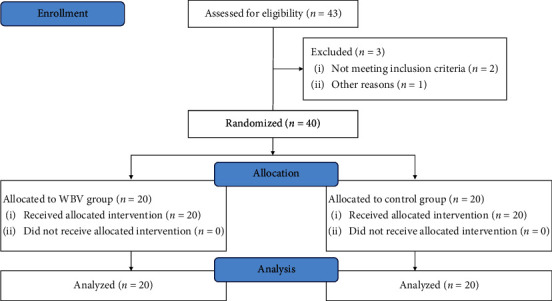
Flowchart of the study. WBV, whole-body vibration.

The participants were recruited through the Internet and posters placed at Shanghai Shangti Orthopaedic Hospital. A total of 40 individuals participated in this study and were randomly allocated to the control group and WBV group (Figure 1). All subjects underwent X-ray or MRI to eliminate specific low back pain, and the clinician performed lumbar function tests assessing lumbar rotation, flexion, and extension. Inclusion criteria were as follows: 18–35 years of age, low back pain persisting for approximately 12 weeks or longer, and at least 3 episodes. Exclusion criteria were as follows: taking analgesic and/or anti-inflammatory agent, previous major trauma and/or surgery of the spine, serious spinal pathology (vertebral fracture, inflammatory arthropathy, spondylolisthesis, rheumatic diseases, cauda equina syndrome, tumor or cancer), cardiovascular and/or neurological disorders, insufficiently treated hypertension, acute inflammation of the musculoskeletal system, and pregnancy. Participants were asked not to change their daily lifestyle and/or to perform additional physical therapy during the study period.

### 2.3. Procedure

After the collection of participant's basic information, the surface electrodes were placed on their bilateral erector spinae (ES), bilateral multifidus (MF), bilateral transversus abdominus/internal oblique (TrA/IO), bilateral rectus abdominis (RA), right deltoid anterior, and right deltoid middle. Forty individuals were randomly allocated to the control group and WBV group. The WBV group performed a 3-minute warm-up, 15-minute WBV training, and 3-minute cool-down exercise. The control group only performed the 3-minute warm-up and 3-minute cool-down exercise with a 15-minute break. Before and after intervention, sEMG signals of each muscle were recorded during right shoulder flexion and abduction in the standing position for 3 times, and the relative activation time of each muscle was calculated.

### 2.4. Intervention

**Figure 2:**
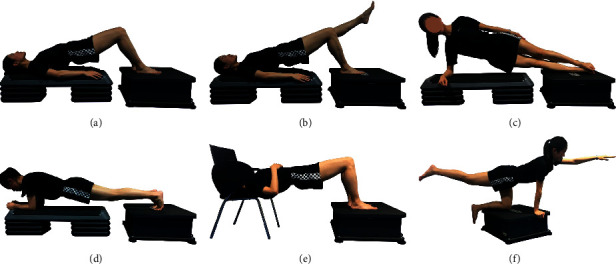
Training program for whole-body vibration exercise. Training program included (a) bridge, (b) bridge with leg lift, (c) side plank, (d) plank, (e) inverse bridge, and (f) balancing table pose.

In the WBV group, all exercises were performed on a vertical vibration instrument (AV009; BODYGREEN, Taiwan, China). Participants were asked to take off their shoes to avoid slowing vibrations on the human body. WBV exercise contains six exercise postures: bridge, bridge with leg lift, side plank, plank, inverse bridge, and balancing table pose. Postures were maintained in two modes (no WBV and WBV) for 20 s and repeated twice with 15 s of rest. The vibration frequency was 20 Hz, and the amplitude was 2 mm. In clinical practice, these postures are widely used and are safe for patients with LBP.  Figure 2 and Table 1 display more detailed information about the WBV exercise protocol. All exercises were completed under the supervision of registered physical therapists.

In the control group, participants also took off their shoes, rested for 15 minutes after completing the 3-minute warm-up exercise on the same exercise plane and then performed the 3-minute cool-down exercise. During the break, the subject is provided health education by the rehabilitation therapist.

### 2.5. sEMG Recording

Fine sandpaper and alcohol swab are used to abrade and clean the skin. After the skin being dry, pairs of Ag/AgCl surface electrodes were placed to the following sites:

  Right deltoid anterior: The upper Ag/AgCl surface electrode is placed approximately 3 cm below the right clavicle bone, and then, follow the muscle fibers, the lower electrode goes laterally at approximately a 25-degree angle from vertical.
  Right deltoid middle: The two electrodes, 2 cm apart, are placed on the lateral aspect of the right upper arm and approximately 3 cm below the acromion, and run parallel to the muscle fibers.
  ES: Place the first electrode piece 2 cm laterally from the spinous process of L1 and the other piece upward.
  MF: Connect the posterior superior iliac spine to the center of L1/L2 vertebrae, place the electrodes at the intersection of horizontal line along the L5 vertebra [35].
  TrA/IO: Place electrodes about 2 cm inferior and medial to the anterior superior iliac spine. This area is bounded inferiorly by the inguinal ligament and RA, and is below the external oblique fibers.
  RA: Electrodes placed 2 cm lateral to the mid-line and 3 cm upward to the umbilicus [36].

The weight-shifting task: the participant stood naturally with their feet shoulder width apart and arms naturally drooping, a 10-pound [37] dumbbell in their right hand and 5-pound in their left hand to stabilize the trunk. When the EMG signal of each muscle were observed to be stable, the participant was given a verbal cue to make their right shoulder flexion to 170° or make their right shoulder abduction to 170° as quickly as possible. The participant should try to avoid trunk rotation and shrug during the right-arm movement. Before and after intervention, the standing shoulder flexion and abduction test was repeated 3 times. Furthermore, to minimize the impact on participant anticipation of the verbal cue, a random time interval between verbal cues was set up.

### 2.6. Data Processing

The sEMG data were collected by Noraxon TeleMyo 2400 DTS system (Noraxon, Inc., USA) and processed by MATLAB 2016a (The Mathworks, USA). Raw sEMG signals sampled at 1500 Hz performed band-pass filtered between 10 and 500 Hz. Subsequently, proceed to full-wave rectification. Then, there are three steps to process data for reflecting muscles' temporal firing pattern. First, a threshold value was calculated by two standard deviations from the mean value of first 400 frames of each sEMG channel. Second, determine the onset moment of muscle activity. That moment, named muscle onset time, was defined as the time when the sEMG signal beyond its threshold for a period of 50 ms [38, 39]. Third, the relative differences in the muscle onset times between the prime mover (i.e., the deltoid) and each trunk muscles (i.e., the ES, MF, RA and TrA/IO) were calculated [40]. The onset time difference between the prime mover and each muscle was calculated by the following equation:

(1) $$\begin{array}{c} \text{target muscle relative onset time}\left(\text{ms}\right)=\text{target muscle onset time}\left(\text{ms}\right)‐\text{prime mover onset time}\left(\text{ms}\right). \end{array}$$

Correspondingly, a negative value represented that the target muscle activated before the prime mover, and vice versa. In this study, the prime mover for the right shoulder flexion is deltoid anterior, and for right shoulder abduction is deltoid middle. Each onset time not only processed in MATLAB 2016a but also was checked visually to verify that sEMG activation was not ambiguous or misinterpreted by movement artefact.

### 2.7. Statistical Analysis

SPSS 20.0 and Microsoft Excel 2016 were used for data logging and statistical analysis. Demographic data were collected for descriptive statistics, which are described as mean ± standard deviation (SD). The data were tested for normality using the Shapiro–Wilk test. The independent-samples *t*-test was used to compare the demographic data of the WBV group and control group. Each subject was required to complete 3 times right shoulder flexion and 3 times right shoulder abduction before and after the intervention. The relative onset time of each muscle was calculated and averaged, named relative activation time. The paired-samples *t*-test was used to compare relative activation time before and after intervention, and independent-samples *t*-test was used to compare the difference of relative activation time between two groups. Significance level was set as *P* < 0.05.

## 3. Results

### 3.1. Demographics Data

20 NSLBP patients average aged 23.6 years old in the WBV group and 20 NSLBP patients average aged 24.2 in the control group voluntarily participated in this study. Other baseline demographic and clinical characteristics of participants are shown in Table 2. No adverse events were observed by physical therapists or reported by NSLBP patients during and after the intervention.

### 3.2. Comparison of Relative Activation Time between/within Groups on Shoulder Flexion

At the baseline, when flexing the shoulder, the bilateral ES, bilateral MF, bilateral TrA/IO, and bilateral RA in two groups are activated after the prime mover muscles (delta anterior muscle). There was no significant difference in the relative activation time of each muscle among two groups (*P* > 0.05) (Table 3).

Using the independent-samples *t*-test to compare the posttest data, it was found that after intervention, the relative activation time of the right TrA/IO and the left RA in the WBV group was significantly less than that in the control group (right TrA/IO: *t* = −2.901, *P*=0.006; left RA: *t* = −2.135, *P*=0.039). And there was no significant difference in the relative activation time of the remaining muscles between the two groups (*P* > 0.05) (Table 3).

In the WBV group, after a single-section intervention, except for the right ES, the relative activation time of each muscle decreased, and the relative activation time of bilateral MF and bilateral TrA/IO was significantly reduced (right MF: *t* = 2.717, *P*=0.014; left MF: *t* = 2.828, *P*=0.011; right TrA/IO: *t* = 2.951, *P*=0.008; left TrA/IO: *t* = 2.407, *P*=0.026). The relative activation time of the right ES after intervention increased slightly, but there was no significant difference compared with baseline (*t* = −0.159, *P*=0.875). In control group, there was no significant difference in the relative activation time of each muscle before and after the intervention (*P* > 0.05) (Table 3).

### 3.3. Between-Group Comparison of Variation in Relative Activation Time on Shoulder Flexion

**Figure 3:**
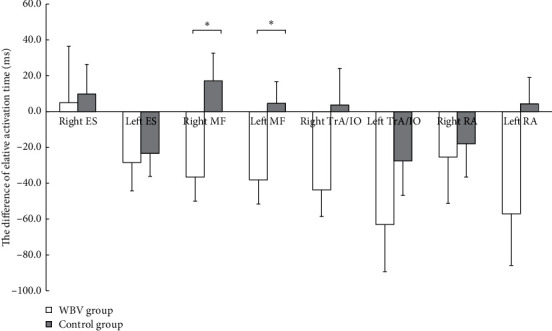
Between-group comparison of variation in relative activation time on shoulder flexion. Variation = post-test–pre-test, a negative value indicated that the intervention shortened the relative activation time, and vice versa. WBV, whole-body vibration; ES, erector spinae; MF, multifidus; TrA/IO, transversus abdominus/internal oblique; RA, rectus abdominis. The independent-samples *t*-test was used to compare the variation. ^*∗*^Significant at *P* < 0.05.

Using the independent-samples *t*-test to compare the variation (Δ = posttest-pretest) in the relative activation time between the two groups after the intervention, it was found that the change value in the relative activation time of the bilateral MF in the WBV group was significantly smaller than that in the control group (right MF: *t* = −2.622, *P*=0.013; left MF: *t* = −2.359, *P*=0.024). There was no significant difference in other muscles (Figure 3).

### 3.4. Comparison of Relative Activation Time between/within Groups on Shoulder Abduction

At baseline, when making the upper limbs abduction, the bilateral ES, bilateral MF, bilateral TrA/IO, and bilateral RA in two groups are activated after the prime mover muscles (delta middle muscle). There was no significant difference in the relative activation time of each muscle among two groups (*P* > 0.05) (Table 4).

After completing a single section of intervention, the relative activation time of each muscle in participants of the WBV group decreased. Except for the left TrA/IO and the left RA, the relative activation time of other muscles was significantly reduced (right ES: *t* = 3.847, *P*=0.001; left ES: *t* = 4.641, *P* < 0.001; right MF: *t* = 4.093, *P*=0.001; left MF: *t* = 2.093, *P*=0.009; right TrA/IO: *t* = 5.239, *P* < 0.001; right RA: *t* = 3.800, *P*=0.001). In the control group, there was no significant difference in the relative activation time of each muscle before and after the intervention (*P* > 0.05).

Using the independent-samples *t*-test to compare the post-test data, it was found that after intervention, the relative activation time of the left ES, right MF, right TrA/IO and right RA in the WBV group was significantly less than that in the control group (left ES: *t* = −3.283, *P*=0.002; right MF: *t* = −2.552, *P*=0.015; right TrA/IO: *t* = −3.113, *P*=0.004; right RA: *t* = −3.984, *P* < 0.001). The relative activation time measured by the remaining muscles after intervention in the WBV group was slightly reduced compared with the control group, however, with no significant difference (*P* > 0.05) (Table 4).

### 3.5. Between-Group Comparison of Variation in Relative Activation Time on Shoulder Abduction

**Figure 4:**
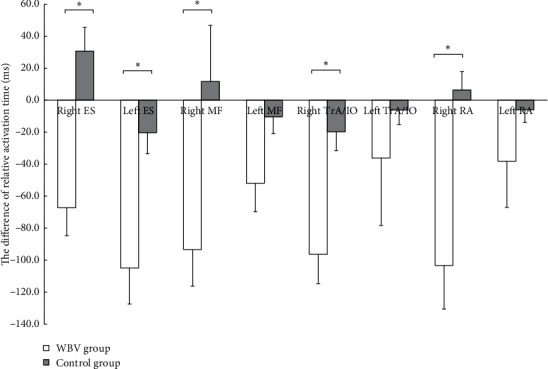
Between-group comparison of variation in relative activation time on shoulder abduction. Variation = post-test–pre-test, a negative value indicated that the intervention shortened the relative activation time, and vice versa. WBV, whole-body vibration; ES, erector spinae; MF, multifidus; TrA/IO, transversus abdominus/internal oblique; RA, rectus abdominis. The independent-samples *t*-test was used to compare the variation. ^*∗*^Significant at *P* < 0.05.

Using the independent samples *t*-test to compare the variation (Δ = posttest-pretest) in the relative activation time between the two groups after the intervention. It was found that the change value in the relative activation time of the bilateral ES, right MF, and right RA in the WBV group was significantly smaller than that in the control group (right ES: *t* = −4.274, *P* < 0.001; left ES: *t* = −3.234, *P*=0.003; right MF: *t* = −2.514, *P*=0.016; right TrA/IO: *t* = −3.518, *P*=0.001; right RA: *t* = −3.717, *P*=0.004). There was no significant difference in other muscles (Figure 4).

## 4. Discussion

The present study's main objective was to evaluate the effects of a single-section WBV exercise on the activation time of core muscles in NSLBP patients. The results of this study demonstrate that (1) WBV exercise shorten the activation time of bilateral MF and bilateral TrA/IO on standing shoulder flexion task, which means the deep core muscles tend to be much easier to activated in maintain the sagittal balance after WBV exercise; (2) WBV exercise shorten the activation time of bilateral ES, bilateral MF, right TrA/IO, and right RA on the standing shoulder abduction task, which means core muscles in lumbar and right abdomen tend to be much easier to activated in maintain the coronal balance after WBV exercise. In addition, with an eye to vibration in relative activation time, MF's relative activation time is significantly shortened by WBV exercise no matter in shoulder flexion or abduction.

Previous literature has many different studies on muscle activation time. Based on the weight shift task, the current studies stated that the onset of the sEMG activity of all trunk muscles occurred prior to that of the muscle in charge of limb movement in healthy individuals [5, 37]. This phenomenon contributes to the feedforward postural response. Furthermore, the anticipatory activation of trunk muscles (e.g., TrA, ES and MF), known as APAs, is vital to maintain lumbopelvic stability during predictable postural perturbations, just as those turned up during limb-oriented movements [4, 41]. APAs counteract the predictable intrinsic reactive forces induced by a focal movement through preactivation of particular muscle groups [42]. Multiple studies demonstrated low back pain patients have shown anticipatory delays in the TrA/IO and MF during postural tasks [8, 43–45]. Hodges [46] claimed that delays in anticipatory muscle activation might be a central nervous system adaptation to pain. Also, Hungerford suggested that the delay in anticipatory muscle is associated with failure of lumbopelvic stabilization [47]. These results are in line with our study. We tested the relative activation muscle by the weight-shifting task (shoulder flexion and abduction) for NSLBP patients. Before intervention, irrespective of the WBV group or control group, the trunk muscles of ES, MF, TrA/IO, and RA showed a positive value of relative activation time, which means ES, MF, TrA/IO, and RA were fired after deltoid. Anticipatory delays were observed. Furthermore, previous studies focused on the activation time of deep fiber like MF and TrA; our study provided available information about the trunk muscle containing ES and RA to supply APA delays in NSLBP patients.

As a noninvasive therapy method, WBV exercise acts like a mild exercise on the body [48, 49]. In recent years, WBV exercise are performed for wild range of patients with metabolic syndrome [50, 51] and musculoskeletal problems including low back pain [33], knee osteoarthritis [52, 53], fibromyalgia [54], osteogenesis imperfecta [55], and so on. Although our research focused on the relative activation time of trunk muscles for APAs in lumbar stability, the intrinsic value of coactivation of core muscles for maintaining lumbopelvic stabilization has been recognized in clinical knowledge. In our two previous studies, we investigated the effect of 12-week WBV exercise in young adults with NSLBP; the results showed that WBV exercise improved lumbar flexion and extension proprioception and reduced pain [56]; then, the sEMG root mean square was used to measure the core muscle activity influenced by WBV exercise in healthy young adults. The results shows that plank, bridge with leg lift, and single plank can fully activate MF, ES, IO, and RA [57]. Based on these studies, we designed this experiment to explore whether WBV exercise alleviates anticipatory delays on the trunk muscle, leading an enhancement clinical performance for NSLBP patients. Our findings that WBV exercise shortens the activation time differently in different shoulder movement may bolster this point.

Our study has several limitations. First, this research focuses on investigating sEMG onset activities of trunk muscles after a single-section WBV exercise, and we recruited a relatively small group of NSLBP patients. Hence, our findings might be cautious to popularize for the entire population with NSLBP. Second, the patients were recruited from different ways, so they have different educational backgrounds, personalities, economic status, and so on. These biopsychosocial factors may affect the patients' symptom after WBV intervention. In addition, a band-pass filter was applied to minimize relevant artifacts in every sEMG collection, but it unavoidably eliminated the actual muscle activity signals. To make impartial contrasts, every muscle activity signal in our study performed the same filtering process. Finally, every participant received only single-section WBV exercise; the effect of the long-term intervention should be performed in further study. Nevertheless, this study offers a reasonable proposal for training programs about WBV exercise, extending the knowledge about possible progressions to improve lumbar stability and muscle function, that is, WBV may shorten the activation time to improve APAs in NSLBP patients.

## 5. Conclusions

In conclusion, single treatment of WBV exercise can effectively alleviate the delayed activation of core muscles in NSLBP patients, but the long-term effects still need further study.

## Figures and Tables

**Table 1:** Parameters and intensity of whole-body vibration exercise in WBV group.

Exercise program	No vibration	Vibration (20 Hz, 2 mm)	Repetitions (times)
Bridge	20 s, interval 15 s	20 s, interval 15 s	2
Bridge with leg lift	20 s, interval 15 s	20 s, interval 15 s	
Side plank	20 s, interval 15 s	20 s, interval 15 s	
Plank	20 s, interval 15 s	20 s, interval 15 s	
Inverse bridge	20 s, interval 15 s	20 s, interval 15 s	
Balancing table pose	20 s, interval 15 s	20 s, interval 15 s	

**Table 2:** Demographic and clinical characteristics of participants.

	WBV group (*n* = 20)	Control group (*n* = 20)	*t* value	*P* value†
Age (y)	23.6 ± 3.3	24.2 ± 2.4	−0.721	0.475
Height (cm)	168.8 ± 7.7	169.1 ± 9.5	−0.110	0.913
Weight (kg)	64.83 ± 13.18	63.88 ± 13.24	0.227	0.821
BMI (kg/m^2^)	22.53 ± 3.10	22.11 ± 2.82	0.454	0.652
Time since first experience with NSLBP (mo)	50.8 ± 45.0	28.9 ± 24.5	1.911	0.064
VAS max	4.40 ± 1.57	4.75 ± 1.55	−0.709	0.483
VAS mean	2.65 ± 0.81	2.75 ± 0.97	−0.354	0.725

WBV, whole-body vibration; BMI, body mass index (calculated as weight in kilograms divided by height in meters squared); NSLBP, nonspecific low back pain; VAS, visual analogue scale. Values are expressed as mean ± SD.^†^Analyzed by the independent-samples *t*-test.

**Table 3:** Comparison of relative activation time between/within groups on shoulder flexion ($\bar{x}$ ± *s*, unit: ms).

		WBV group (*n* = 20)	Control group (*n* = 20)	WBV – Control (95% CI)	*P*^†^ value
Right ES	Pre-test	41.5 ± 156.5	61.8 ± 125.5	−20.3 (−111.1 to 70.5)	0.653
	Post-test	46.5 ± 139.8	71.5 ± 94.6	−25.1 (−101.4 to 51.3)	0.511
	*P*^‡^ value	0.875	0.564		
Left ES	Pre-test	34.0 ± 94.5	21.7 ± 95.2	12.2 (−48.5 to 72.9)	0.686
	Post-test	5.6 ± 71.9	−1.6 ± 68.7	7.2 (−37.8 to 52.2)	0.748
	*P*^‡^ value	0.089	0.083		
Right MF	Pre-test	85.2 ± 94.4	54.6 ± 103.7	30.6 (−32.9 to 94.1)	0.335
	Post-test	48.6 ± 78.7	71.1 ± 98.2	−23.1 (−80.0 to 33.9)	0.418
	*P*^‡^ value	0.014^*∗*^	0.281		
Left MF	Pre-test	68.3 ± 81.9	52.3 ± 99.0	16.0 (−42.1 to 74.2)	0.580
	Post-test	30.2 ± 81.1	56.9 ± 95.1	−26.7 (−83.3 to 29.9)	0.346
	*P*^‡^ value	0.011^*∗*^	0.710		
Right TrA/IO	Pre-test	147.2 ± 79.9	202.2 ± 94.0	−55.1 (−110.9 to 0.8)	0.053
	Post-test	103.4 ± 96.4	205.9 ± 125.1	−102.5 (−174.0 to −31.0)	0.006^*∗*^
	*P*^‡^ value	0.008^*∗*^	0.860		
Left TrA/IO	Pre-test	160.3 ± 119.3	222.8 ± 168.2	−62.5 (−155.9 to 30.8)	0.183
	Post-test	97.2 ± 159.0	195.2 ± 147.9	−98.0 (−196.3 to 0.3)	0.051
	*P*^‡^ value	0.026^*∗*^	0.167		
Right RA	Pre-test	259.4 ± 137.1	236.1 ± 162.2	23.3 (−72.9 to 119.4)	0.627
	Post-test	234.0 ± 118.1	218.2 ± 156.7	15.8 (−73.0 to 104.6)	0.721
	*P*^‡^ value	0.337	0.346		
Left RA	Pre-test	231.4 ± 155.0	267.7 ± 132.3	−36.4 (−128.6 to 55.9)	0.430
	Post-test	174.3 ± 147.8	272.0 ± 141.7	−97.8 (−190.4 to −5.1)	0.039^*∗*^
	*P*^‡^ value	0.062	0.772		

Relative activation time = muscle onset time-prime mover onset time (ms), a negative value indicated that the target muscle fired before the prime mover, and vice versa. WBV, whole-body vibration; ES, erector spinae; MF, multifidus; TrA/IO, transversus abdominus/internal oblique; RA, rectus abdominis; CI, confidence interval. Values are expressed as mean ± SD.^†^Analyzed by the independent-samples *t*-test;^‡^analyzed by the paired-samples *t*-test;^*∗*^significant at *P* < 0.05.

**Table 4:** Comparison of relative activation time between/within groups on shoulder abduction ($\bar{x}$ ± *s*, unit: ms).

		WBV group (*n* = 20)	Control group (*n* = 20)	WBV – Control (95% CI)	*P*^‡^ value
Right ES	Pre-test	184.8 ± 99.8	147.3 ± 159.9	37.5 (−47.8 to 122.8)	0.379
	Post-test	117.5 ± 115.4	178.0 ± 130.8	−60.4 (−139 to 18.5)	0.130
	*P*^‡^ value	0.001^*∗*^	0.052		
Left ES	Pre-test	162.5 ± 108.8	158.0 ± 104.2	4.5 (−63.7 to 72.7)	0.894
	Post-test	57.7 ± 76.7	137.8 ± 77.5	−80.1 (−129.4 to −30.7)	0.002^*∗*^
	*P*^‡^ value	0.000^*∗*^	0.141		
Right MF	Pre-test	237.5 ± 97.4	257.6 ± 100.2	−20.1 (−83.4 to 43.1)	0.524
	Post-test	144.1 ± 97.1	269.4 ± 196.9	−125.3 (−224.7 to −25.9)	0.015^*∗*^
	*P*^‡^ value	0.001^*∗*^	0.741		
Left MF	Pre-test	157.3 ± 90.3	163.0 ± 76.3	−5.7 (−59.2 to 47.8)	0.830
	Post-test	105.4 ± 108.9	152.6 ± 81.2	−47.2 (−108.6 to 14.3)	0.129
	*P*^‡^ value	0.009^*∗*^	0.333		
Right TrA/IO	Pre-test	165.7 ± 100.4	186.3 ± 122.5	−20.6 (−92.6 to 51.1)	0.564
	Post-test	69.4 ± 102.9	166.5 ± 94.4	−97.2 (−160.4 to −34.0)	0.004^*∗*^
	*P*^‡^ value	0.000^*∗*^	0.105		
Let TrA/IO	Pre-test	160.7 ± 143.4	195.1 ± 108.6	−34.5 (−115.9 to 47.0)	0.397
	Post-test	124.6 ± 206.6	189.0 ± 100.4	−64.5 (−168.4 to 39.5)	0.217
	*P*^‡^ value	0.403	0.508		
Right RA	Pre-test	231.3 ± 129.6	258.9 ± 123.1	−27.6 (−108.5 to 53.3)	0.494
	Post-test	128.0 ± 99.3	265.3 ± 117.9	−137.3 (−207.1 to −67.5)	0.000^*∗*^
	*P*^‡^ value	0.001^*∗*^	0.584		
Left RA	Pre-test	108.8 ± 218.6	166.1 ± 181.9	−57.4 (−186.1 to 71.3)	0.373
	Post-test	70.6 ± 127.7	160.1 ± 176.6	−89.5 (−188.1 to 9.2)	0.074
	*P*^‡^ value	0.203	0.452		

Relative activation time = muscle onset time-prime mover onset time (ms), a negative value indicated that the target muscle fired before the prime mover, and vice versa. WBV, whole-body vibration; ES, erector spinae; MF, multifidus; TrA/IO, transversus abdominus/internal oblique; RA, rectus abdominis; CI, confidence interval. Values are expressed as mean ± SD.^†^Analyzed by the independent-samples *t*-test;^‡^ analyzed by the paired-samples *t*-test;^*∗*^ significant at *P* < 0.05.

## Data Availability

The data used to support the findings of this study are included within the article.
